# Development of predictive models for the prognosis of triple-negative breast cancer using multiple transcriptomic analyses

**DOI:** 10.1371/journal.pone.0348414

**Published:** 2026-05-04

**Authors:** Suhyun Hwangbo, Yoojin Choi, Jae Yong Ryu

**Affiliations:** 1 Department of Genomic Medicine, Seoul National University Hospital, Seoul, Republic of Korea; 2 Department of Chemical and Biological Engineering, Chungwoon University, Incheon, Republic of Korea; 3 School of Systems Biomedical Science, Soongsil University, Seoul, Republic of Korea; Qatar Biomedical Research Institute, QATAR

## Abstract

Triple-negative breast cancer (TNBC) is a subtype of breast cancer (BC) and constitutes approximately 15–20% of all BC cases. This subtype has the most aggressive behavior and the worst prognosis. Numerous studies have been conducted over the past several decades to address the lack of clinically available treatment options. In particular, potential markers targeting effective treatment options have been actively studied. However, these efforts were hindered by the complex mechanisms of TNBC, and no study has demonstrated a model with a predictive performance exceeding 0.85. This study developed TNBC prognosis predictive models with a predictive performance exceeding 0.94. Applying the nine selected markers to five independent datasets demonstrated their potential as TNBC-specific prognostic markers. Most of these genes (including *GPR61*, *PZP*, *IGFL1*, and *AHCTF1*) are associated with overall survival (OS) in patients with TNBC. Based on these results, these nine selected genes may serve as prognostic markers for OS in patients with TNBC.

## Introduction

Breast cancer (BC) is the most commonly diagnosed cancer in women worldwide and has the highest cancer-related mortality rate [1,2]. It is heterogeneous and is generally classified into four subtypes based on the expression of the estrogen receptor (ER), progesterone receptor (PR), and human epidermal growth factor receptor-2 (HER2) [2,3]. Triple-negative breast cancer (TNBC) is one of the four subtypes of BC, accounting for approximately 15–20% of all BC cases [2,4]. It is defined as the absence of the expression of three receptors (ER, PR, and HER2) [5], resulting in unresponsiveness to agents targeting hormone receptors and HER2. Significant progress has been made in addressing this issue, particularly with novel agents such as poly ADP-ribose polymerase inhibitors and antibody-drug conjugates for patients with TNBC [6–8]. However, these studies have primarily focused on subsets of patients with TNBC, such as those with germline mutations in BRCA1/2 or metastatic TNBC.

Despite these advancements, TNBC remains a challenging disease with poorer outcomes compared to other BC subtypes. Specifically, the 5-year overall survival (OS) rate of primary TNBC is significantly lower (77%) than that of other BC subtypes (93%): Luminal A (LumA), Luminal B (LumB), and HER2-positive [9–11]. Given this poor prognosis and the lack of effective targeted therapies for most TNBC patients, identifying robust prognostic biomarkers has important clinical implications. Such biomarkers can improve risk stratification, guide treatment decisions, and identify patients who may benefit from novel therapeutic strategies or intensified surveillance [12]. Thus, there is a critical need for additional prognostic markers and therapeutic targets to further improve patient outcomes, which is the primary aim of this study.

Numerous studies have been conducted over the past several decades to overcome the limitations of treatment options for patients with TNBC by identifying potential markers for targeting effective treatment options. Neoadjuvant chemotherapy (NAC) aimed at reducing tumor size has been established as the standard treatment for TNBC, with improved prognosis for patients achieving a pathological complete response (pCR) [2,13]. Owing to the clinical benefits of NAC, many studies have focused on identifying the markers that predict the pCR after NAC to stratify patients with more effective NAC responses [10,14,15]. Studies have also identified predictors of sensitivity to chemotherapy in TNBC (such as *BCL2*), including postoperative adjuvant chemotherapy [16,17]. Additionally, predictors of the response to immune checkpoint inhibitors (ICI) plus chemotherapy were conducted to identify new therapeutic agents [18,19]. Unfortunately, these efforts failed, and effective treatment strategies have not yet been developed owing to the complex mechanisms of TNBC that do not solely rely on specific signals [20].

In addition to chemotherapy or ICI, previous studies have attempted to identify prognostic markers that predict outcomes, such as recurrence, death, disease-free survival (DFS), and OS, in patients with TNBC [5,21,22]. Campione et al. developed a model to predict recurrence using three protein signatures (*TrpRS*, *TSP1*, and *DP*), achieving predictive performance with an area under the curve (AUC) of 0.82 [5]. Xu et al. developed predictive models for death based on multiple machine learning algorithms using clinicopathological data, with the highest predictive performance, achieving an AUC of 0.732 [21]. Yang et al. developed a nomogram to predict DFS and OS using clinicopathological data, with the developed nomogram displaying a predictive performance of AUC 0.784 for DFS and AUC 0.783 for OS, respectively [22]. However, there is no current model with a predictive performance surpassing 0.9 on validation data, indicating the ongoing necessity for developing models with greater accuracy.

Recently, research has been conducted to identify TNBC markers using data from LumA, which expresses two hormone receptors and has the best prognosis among the BC subtypes. Choi et al. constructed a molecular regulatory network model for reprogramming TNBC cells into LumA cells and identified *BCL11A* and *HDAC1/2* as the optimal targets for inducing the transition to LumA cells (1). Singhal et al. established TNBC cell line-driven *SLFN12*-overexpressing human BC xenografts that led to higher levels of LumA markers, HER2 receptor expression, and ultimately better survival [23].

In this study, we aimed to develop predictive models with outstanding performance in forecasting the prognosis of TNBC subtypes by employing multiple machine learning algorithms and identifying TNBC-specific prognostic markers through validation using multi-cohort transcriptomic datasets.

## Materials and methods

### Data sources

This retrospective study used six datasets: four RNA-sequencing (RNA-seq) datasets and two microarray datasets. The RNA-seq dataset from The Cancer Genome Atlas Breast Invasive Cancer (TCGA-BRCA) cohort served as the primary dataset for model development; gene expression profiles (Illumina HiSeq 2000) were obtained from the UCSC Xena Data Hub (https://xenabrowser.net/). Only primary tumors were included to build a prognostic model for early outcome prediction. Subtypes were defined by the Prediction Analysis of Microarray 50 (PAM50) signature and comprised 143 TNBC (basal-like), 386 LumA, 186 LumB, and 69 HER2-positive samples. OS and death events were used as the primary outcomes.

For external validation of prognostic genes identified during model development, we used two Gene Expression Omnibus (GEO) datasets (GSE65216 and GSE215442), two cell-line datasets, and the METABRIC cohort. GSE65216 is a GPL570 (Affymetrix U133 Plus 2.0) microarray dataset including 55 TNBC, 29 LumA, 30 LumB, and 39 HER2-positive samples. GSE215442 is an RNA-seq dataset generated from MDA-MB-231 TNBC cells overexpressing *SLFN12* to create LumA-like subclones with favorable prognosis, comprising three *SLFN12*-overexpressing lines and three controls [23]. The cell-line datasets consisted of a single-cell RNA-seq dataset (7,484 TNBC and 4,599 LumA cells) and a bulk RNA-seq dataset (31 TNBC and 10 LumA cell lines). METABRIC is a microarray cohort of 320 TNBC tumors used for external validation; during follow-up, 168 patients (52%) died, with a median OS of 13.3 years.

### Model development and evaluation

**Fig 1:**
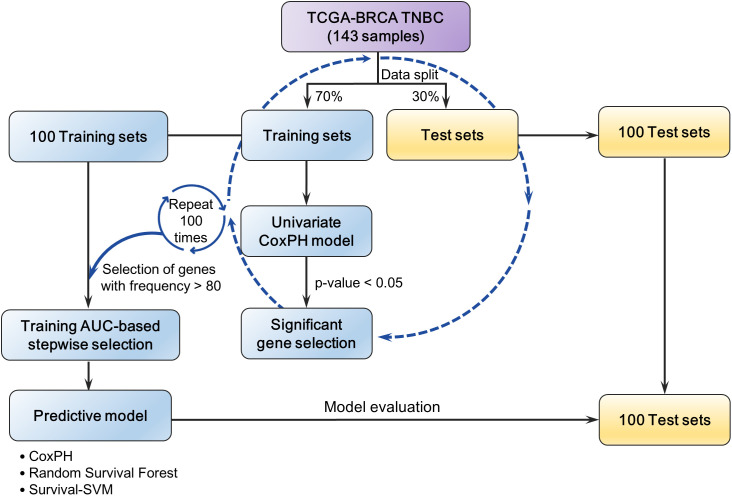
Workflow for model development and evaluation. The dataset was randomly split 70/30 into training and test sets. Across 100 resamples, univariate Cox proportional hazards models were fit in the training set; genes significant in ≥80/100 resamples were retained as candidates. An AUC-based stepwise selection in the training set produced the final gene signature. For each resample, the signature was trained on the training set and evaluated on its paired test set, and performance (AUC) was summarized across all 100 pairs. Outcomes were 5-year and 10-year overall survival.

The overall workflow is illustrated in Fig 1. The objective of this study was to develop a prognostic model for TNBC using the Cox proportional hazards (CoxPH) regression based on time-to-event data. All 143 TNBC samples from the TCGA-BRCA cohort were included, since CoxPH models estimate relative risk within the cohort without requiring a predefined control group.

The dataset was randomly divided into training (70%) and test (30%) subsets, stratified by event status to maintain the proportion of deaths and censored cases. For each gene, a univariate CoxPH model was fitted using the training set, and genes with p-value < 0.05 were considered significant. This process was repeated 100 times with random resampling, and genes identified as significant in at least 80 of the 100 iterations were retained as candidate predictors.

To determine the optimal combination of predictors, we applied a training AUC-based stepwise selection procedure. Each candidate gene was first fitted individually using CoxPH, and the mean training AUC across 100 resamples was calculated. The gene with the highest mean AUC was chosen as the initial model (M_k_ = M_1_; k = 1).

In each subsequent forward step (k = k + 1), all candidate models formed by adding exactly one previously unselected predictor to the current model (M_k-1_) were evaluated, and the highest-mean-AUC model was designated as M_k_. A new model (M_k_) was accepted only if its mean AUC exceeded that of M_k-1_ by more than α = 0.005, which served as a minimal improvement threshold to prevent overfitting from marginal gains; otherwise, M_k-1_ was retained and the procedure stopped.

In each backward step (k = k + 1), we evaluated all reduced models formed by removing exactly one predictor from M_k-1_ (one-at-a-time deletions; the number of candidates equals the number of predictors in M_k-1_). The reduced model with the highest mean training AUC (M_k_) was retained only if its performance exceeded that of M_k-1_ by more than α = 0.005; otherwise, M_k-1_ was retained and the algorithm returned to the forward step. Forward and backward steps were alternated until no further increase in mean training AUC was observed during the forward phase.

The final predictors obtained through this selection procedure were used to develop prognostic models with three algorithms―CoxPH, Random Survival Forest (RSF), and Survival Support Vector Machine (Survival-SVM). For RSF and Survival-SVM, hyperparameters were optimized by maximizing the training AUC. Model performance across 100 resamples was evaluated using the corresponding test sets, with time-dependent AUC, area under the precision-recall curve (AUPRC) and c-index as performance metrics for 5-year and 10-year overall survival outcomes.

### Statistical analysis

Group differences in gene expression were tested with two-sided Wilcoxon rank-sum tests. For survival analyses, optimal expression cutoffs for each gene were determined using maximally selected rank statistics (MaxStat) [24], after which samples were dichotomized into high- and low-expression groups. Survival differences were compared with two-sided log-rank tests. Associations between expression group (high vs. low) and overall survival status were evaluated using Fisher’s exact test. Statistical significance was set at p-value < 0.05.

## Results

### Development and evaluation of TNBC prognosis model

Among the 16,336 protein-coding genes in the TCGA-BRCA cohort, we initially screened candidate predictors (genes) associated with OS. Of the 784 BC patients in the cohort, 143 (18%) were identified as having TNBC (S1 Table). Among them, NAC history was available for 142 patients, all of whom had no such history; one patient had missing data. During the follow-up period, 18 TNBC patients (13%) died, with a median OS of 20.4 years. To prevent overfitting during screening to model development, the total dataset was randomly divided into training and test sets in a 7:3 ratio, and the process from screening to model development was performed using only the training set. A CoxPH model was used to screen the candidate predictors. The genes were selected at a significance level of 5%. To avoid the selection of specific dataset-dependent predictors, the data was randomly split 100 times to select significant genes. This is expected to reduce the selection bias owing to random splitting. After 100 iterations, 53 predictor variables that were selected as key variables more than 80 times were selected as final candidates.

**Fig 2:**
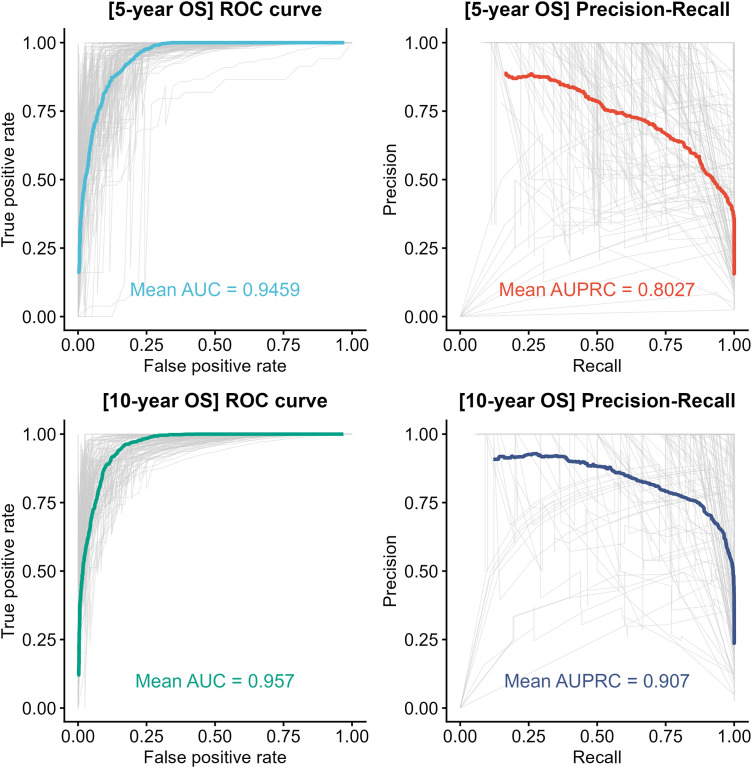
ROC and precision-recall curves of the developed model across 100 training/test splits. For each outcome (5-year OS and 10-year OS), receiver operating characteristic (ROC) and precision-recall curves from 100 resampled test sets are shown as dashed lines, and the mean curves are shown as solid lines. The mean AUC and mean AUPRC are reported for the ROC and precision-recall curves, respectively.

We performed training AUC-based stepwise selection using these 53 candidates. The 5-year OS and 10-year OS (which were used as the main outcomes of the previously developed TNBC prognosis prediction model [22,25]) were used as response variables. For the 5-year OS, over 90% of the 100 test sets achieved a test AUC greater than 0.9, with mean AUC and AUPRC values of 0.9459 and 0.8027, respectively (Fig 2). For the 10-year OS, more than 98% of the test sets achieved a test AUC greater than 0.9, with mean AUC and AUPRC values of 0.9570 and 0.9070, respectively.

In addition to the CoxPH model used as the main model in this study, other machine learning (ML) algorithms (RSF and Survival-SVM) were applied to 100 training and test sets. Both CoxPH and other ML algorithms confirmed predictive performance over 0.8 for AUC, AUPRC, and C-index (Table 1).

**Table 1:** Final selected predictive model for each response variable.

Response variable	No. of predictors	Model	Prediction Measure	ML Algorithm	Training (mean±s.d.)	Test (mean±s.d.)
5-year OS	6	*CELF6* + *IGFL1* + *GPR61* + *TMEM14B* + *CREB5* + *AHCTF1*	AUC	CoxPH	0.9750 ± 0.0087	0.9459 ± 0.0478
				RSF	0.7778 ± 0.0471	0.8024 ± 0.0736
			AUPRC	CoxPH	0.8994 ± 0.0803	0.8027 ± 0.2177
				RSF	0.9894 ± 0.0076	0.7063 ± 0.2537
			C-index	CoxPH	0.9541 ± 0.0121	0.9233 ± 0.0574
				RSF	0.8454 ± 0.0427	0.8688 ± 0.0728
				Survival-SVM	0.9367 ± 0.0149	0.8873 ± 0.0528
10-year OS	6	*PZP* + *TTLL2* + *IGFL1* + *CELF6* + *SRRD* + *GPR61*	AUC	CoxPH	0.9767 ± 0.0085	0.9570 ± 0.0267
				RSF	0.8505 ± 0.0423	0.8812 ± 0.0643
			AUPRC	CoxPH	0.9899 ± 0.0423	0.9070 ± 0.1571
				RSF	0.9838 ± 0.0087	0.7701 ± 0.1906
			C-index	CoxPH	0.9632 ± 0.0111	0.9426 ± 0.0357
				RSF	0.8567 ± 0.0451	0.8812 ± 0.0715
				Survival-SVM	0.9531 ± 0.0163	0.9165 ± 0.0449

AUC, area under the curve; AUPRC, area under the precision-recall curve; CoxPH, Cox proportional hazards; ML, machine learning; RSF, Random Survival Forest; s.d., standard deviation. SVM, Support Vector Machine.

**Fig 3:**
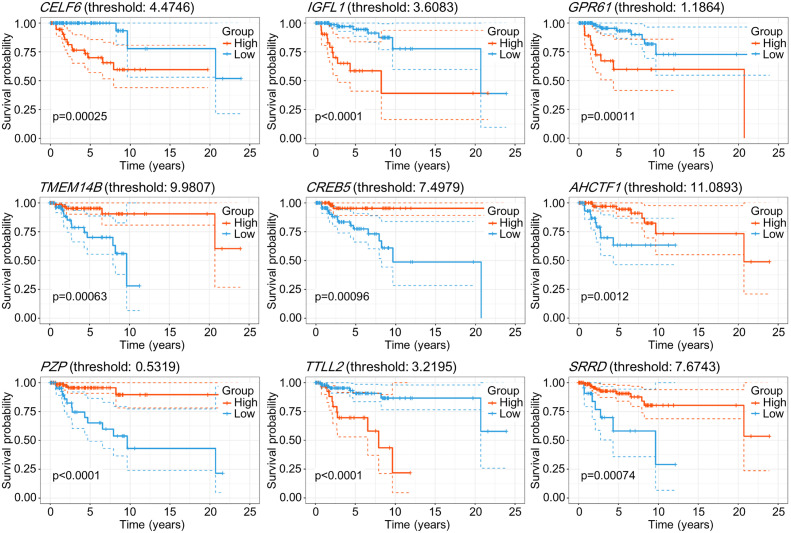
Kaplan-Meier survival curves for nine genes in TNBC (n = 143). For each gene, samples were dichotomized by the MaxStat-derived optimal cutoff into high- (red) and low-expression (blue) groups. Survival differences were evaluated with the log-rank test.

**Fig 4:**
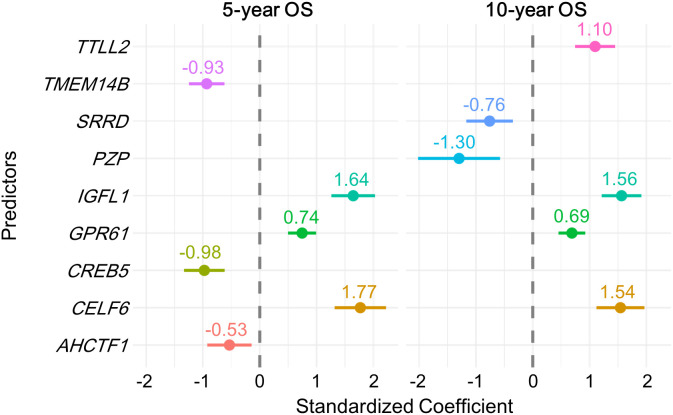
Standardized genes coefficients in OS predictive models. For each outcome (5-year OS and 10-year OS), coefficients from the final model fit to the entire dataset are shown. All variables were z-standardized before fitting to enable direct comparison of gene effects.

The direction and significance of the coefficients contributing to OS showed consistent trends across the 100 training sets and the total dataset. According to the fitted CoxPH models for the entire dataset (Table 2), *CELF6*, *IGFL1*, *GPR61*, and *TTLL2* had positive coefficients, indicating shorter survival with increasing expression levels. In contrast, other predictors (including *TMEM14B* and *CREB5*) showed negative coefficients, indicating shorter survival with decreasing expression levels. These findings were consistent with Kaplan-Meier analyses (Fig 3). In addition, Fisher’s exact test showed significant differences in gene expression group distributions (high vs. low) between survivors and non-survivors (Table 3), further supporting the association between gene expression and survival outcomes. Standardized coefficients indicated that *CELF6* and *IGFL1* were the most influential predictors for OS (Fig 4).

**Table 2:** Fitted results of the CoxPH model for each response variable (multiple analysis).

Response variables	Predictor variables	Hazard Ratio	(95% CI)	p-value
5-year OS	*CELF6*	4.86	(2.20 − 10.74)	9.2E-05
	*IGFL1*	2.15	(1.51 − 3.05)	1.8E-05
	*GPR61*	2.97	(1.46 − 6.04)	0.0026
	*TMEM14B*	0.24	(0.09 − 0.61)	0.0028
	*CREB5*	0.49	(0.30 − 0.82)	0.0064
	*AHCTF1*	0.39	(0.10 − 1.50)	0.1708
10-year OS	*PZP*	0.45	(0.19 − 1.07)	0.0724
	*TTLL2*	2.10	(1.31 − 3.34)	0.0019
	*IGFL1*	2.07	(1.50 − 2.84)	7.8E-06
	*CELF6*	3.97	(1.90 − 8.32)	2.5E-04
	*SRRD*	0.20	(0.04 − 1.10)	0.0641
	*GPR61*	2.74	(1.39 − 5.37)	0.0034

OS, overall survival.

**Table 3:** Association between gene expression groups (high vs. low), defined by gene-specific thresholds, and survival status (alive vs. deceased at follow-up). A Fisher’s exact test was performed to evaluate differences in expression groups between survivors and non-survivors.

Gene	Cutoff	Poor-survival group	Death event rate (%)		p-value
			High expression group	Low expression group	
CELF6	4.4746	High	24.2	3.7	2.9E-04
IGFL1	3.6083	High	30.6	6.5	5.7E-04
GPR61	1.1864	High	34.5	7	3.9E-04
TMEM14B	9.9807	Low	6.2	20.6	1.2E-02
CREB5	7.4979	Low	3	20.8	1.7E-03
AHCTF1	11.0893	Low	8.9	18.9	0.1161
PZP	0.5319	Low	4.3	28.6	7.1E-05
TTLL2	3.2195	High	31.2	7.2	1.0E-03
SRRD	7.6743	Low	9.1	31.8	8.3E-03

### Validation of TNBC prognostic markers across cohorts

**Fig 5:**
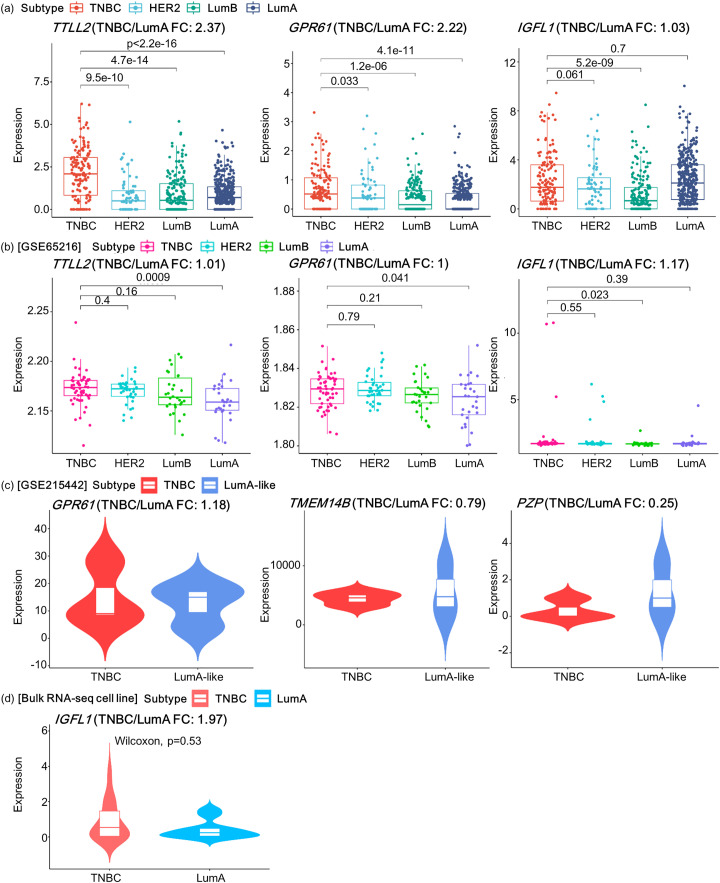
External validation in four independent cohorts. **(a)** TCGA-BRCA; **(b)** GSE65216; **(c)** GSE215442; (d) cell line bulk RNA-seq. For each cohort, boxplots show the genes among the nine selected genes whose expression follows the hypothesized direction. Fold change (FC) is the mean expression in TNBC divided by that in LumA. TNBC was compared with LumA, LumB, and HER2 using two-sided Wilcoxon rank-sum tests.

To confirm the potential of the nine selected prognostic markers for TNBC, we validated them across multiple cohort datasets. We hypothesized that if increased expression levels contributed to a worsening prognosis, its expression level would be higher in TNBC than that in LumA, which is known to have a better prognosis among the BC subtypes. To confirm our hypothesis, we compared the gene expression patterns between TNBC and other BC subtypes for each of the nine genes in the four datasets, including the TCGA-BRCA cohort. Among the nine genes, *TTLL2* and *GPR61* exhibited trends consistent with this hypothesis in both TCGA-BRCA and GSE65216 datasets (Figs 5A and 5B). Specifically, the expression of *TTLL2* and *GPR61* was significantly higher in TNBC than in LumA (p-value < 2.2E-16 and <4.1E-11 for *TTLL2* and *GPR61* in TCGA-BRCA, respectively). Furthermore, the expression levels of both genes were higher in TNBC than those in the other BC subtypes. This trend was consistent for both the genes in the GSE65216 dataset.

Unlike TCGA-BRCA and GSE65216 datasets, the GSE215442 dataset was designed to generate *SLFN12*-overexpressing xenografts from a TNBC cell line (MDA-MB-231), resulting in a LumA-like TNBC cell line with better prognosis. The GSE215442 dataset contained RNA-seq data from two groups: original TNBC and LumA-like TNBC cell lines. Analysis of the GSE215442 dataset identified three genes whose expression level trends in the LumA-like TNBC group compared to those in the original TNBC group were consistent with those of the nine genes selected as predictors (Fig 5C). The *GPR61* gene demonstrated a trend toward decreased survival time with increasing expression levels across TNBC subtypes in TCGA cohort and exhibited higher expression levels in the original TNBC group than those in the LumA-like TNBC group. In contrast, *TMEM14B* and *PZP* displayed a trend toward decreased survival time with decreasing expression levels across the TNBC subtypes in the TCGA cohort and had lower expression levels in the original TNBC group than those in the LumA-like TNBC group. The GSE65216 dataset and the cell-line-based bulk RNA-seq dataset revealed higher *IGFL1* expression levels in TNBC than those in LumA, but the difference was not statistically significant (Figs 5B and 5D).

Through validation analysis of multi-cohort datasets, we confirmed that five genes (*TTLL2*, *GPR61*, *TMEM14B*, *PZP*, and *IGFL1*) were validated in at least one independent dataset. Interestingly, the *GPR61* gene was validated in three datasets, although there is still no known relationship between *GPR61* (G-protein-coupled receptor 61) and TNBC prognosis. *GPR161* (which belongs to the same receptor family as *GPR61*) is overexpressed in TNBC and impairs the proliferation of TNBC cell lines in knockdown experiments [26]. Considering that *GPR161* is a potential drug target, the same is expected for *GPR61*.

## Discussion

We developed prognostic models for TNBC that achieved an AUC exceeding 0.94 in the test sets, outperforming previously reported OS-predictive models, which typically achieved AUCs below 0.85 [22,25,27]. Comparable high-performing models have been reported, including a 10-gene early-stage TNBC signature [28], a stemness-based prognostic model [29], and an EMT-related gene signature [30]. These studies collectively demonstrate that compact gene sets can achieve clinically meaningful risk stratification. Our model extends this approach by incorporating systematic resampling with AUC-based feature selection, thereby improving generalizability.

Beyond technical performance, long-term survival prediction has important clinical relevance. While treatment-response prediction primarily informs initial therapeutic decision-making, long-term survival prediction (5- and 10-year OS) provides complementary but distinct clinical value. TNBC is a highly aggressive and heterogeneous disease, with substantial variability in survival outcomes even among patients with similar clinical characteristics. This heterogeneity necessitates personalized prognostic assessment and accurate long-term risk stratification [31].

Despite extensive research efforts, robust prognostic tools for TNBC remain limited, and existing clinical markers such as pCR provide only partial prognostic information [32]. Survival prediction offers clinically actionable insights beyond treatment response by enabling risk stratification and supporting long-term management decisions, including treatment intensity and follow-up planning [31,33]. In addition, a substantial proportion of TNBC recurrences and deaths occur beyond five years after diagnosis, and even patients who initially achieve favorable responses (e.g., pCR) may experience late relapse [34,35]. Together, these findings indicate that treatment response and long-term survival capture related but distinct aspects of disease progression, underscoring the importance of long-term survival prediction in TNBC.

Among the evaluated algorithms, the CoxPH model showed the best predictive performance (Table 1). Although advanced ML approaches can perform similarly, previous studies have shown that CoxPH-based models remain competitive, and are often superior, when sample sizes are modest and relationships are approximately linear [36,37]. Consistent with these reports, the CoxPH model achieved the highest discrimination in the TCGA-BRCA dataset.

Given the class imbalance in our dataset, AUPRC provides a complementary performance metric to AUC. Only 18 of 142 patients (13%) experienced events, corresponding to a baseline AUPRC of 0.127 for a random classifier. Despite this imbalance, our model achieved AUPRC values of 0.8027 and 0.9070 for 5- and 10-year OS, respectively, substantially exceeding the baseline and demonstrating strong predictive performance for the minority class. The corresponding AUC values were 0.9459 and 0.9570. As AUC and AUPRC have different baselines and scales, direct numerical comparison is not appropriate; however, the consistently high values across both metrics support the robustness of our model under class imbalance.

External validation using the METABRIC cohort yielded lower predictive performance, likely due to differences in clinical composition and assay platforms between METABRIC (microarray) and TCGA-BRCA (RNA-seq). Similar cross-platform discrepancies have been reported, and frameworks such as EMBER have demonstrated that statistical harmonization can improve integration across datasets [38]. Applying such approaches may further enhance cross-cohort reproducibility.

Kaplan-Meier analyses demonstrated consistent survival trends across TCGA-BRCA and METABRIC (S1 Fig), supporting the biological plausibility of the identified markers. Among the nine selected genes, five (*PZP*, *GPR61*, *TTLL2*, *TMEM14B*, and *IGFL1*) showed consistent expression patterns and effect directions across cohorts, in line with prior studies linking them to TNBC proliferation and survival [1,23,26,39,40]. The remaining four genes showed discordant coefficients but similar expression patterns (S2 Fig), suggesting potential subtype-specific effects [41].

Recent studies have identified immune- and B cell-related signatures as strong prognostic determinants in early-stage TNBC [42], suggesting that incorporating immune-related features into our model may further improve predictive performance. In addition to OS, we developed models for progression-free survival (PFS) and DFS using the same framework (S2 Table). As these endpoints reflect distinct biological processes, differences in model performance are expected [43,44]. These findings highlight the potential applicability of our framework across multiple prognostic outcomes.

In summary, we present a reproducible and high-performing prognostic model for TNBC that exceeds prior benchmarks and aligns with emerging literature. Despite limitations in external validation and experimental confirmation, the model’s strong reproducibility across datasets and consistency with recent prognostic studies [28–30,38,45] underscore its robustness. Future studies will focus on experimental validation, cross-platform harmonization, and integration of multi-omics and immune features to further advance precision prognostication in TNBC.

## Supporting information

S1 Fig Kaplan-Meier survival curves for nine genes in the METABRIC cohort (external validation). Analysis followed the procedure in Fig 3, but MaxStat cutoffs were re-estimated within METABRIC for each gene. Samples were dichotomized into high- (red) and low-expression (blue) groups using these cohort-specific cutoffs, and survival differences were assessed with the two-sided log-rank test.
(TIF)

S2 Fig External validation across four independent datasets. (a) TCGA-BRCA; (b) GSE65216; (c) cell line single cell RNA-seq; (d) cell line bulk RNA-seq. Fold change (FC) is the mean expression in TNBC divided by that in LumA. Differences between TNBC and LumA, LumB, and HER2 were tested using two-sided Wilcoxon rank-sum tests.
(TIF)

S1 Table Clinicopathological characteristics of all patients in the TCGA-BRCA cohort. NAC; Neoadjuvant chemotherapy. The p-values for continuous variable (age) and categorical variables were calculated using the Kruskal-Wallis test and the Chi-square test, respectively.
(DOCX)

S2 Table The best model for each of PFS and DFS. PFS: Progression Free Survival; DFS: Disease Free Survival. For the results, we utilized CoxPH as the machine learning algorithm and AUC as the prediction measure, both of which showed the highest predictive performance in Table 1.
(DOCX)
