# Supplementary figures and images for: Development of predictive models for the prognosis of triple-negative breast cancer using multiple transcriptomic analyses
Source: PLoS One. 2026 May 4;21(5):e0348414. doi: 10.1371/journal.pone.0348414 (PMC13138617; doi:10.1371/journal.pone.0348414)

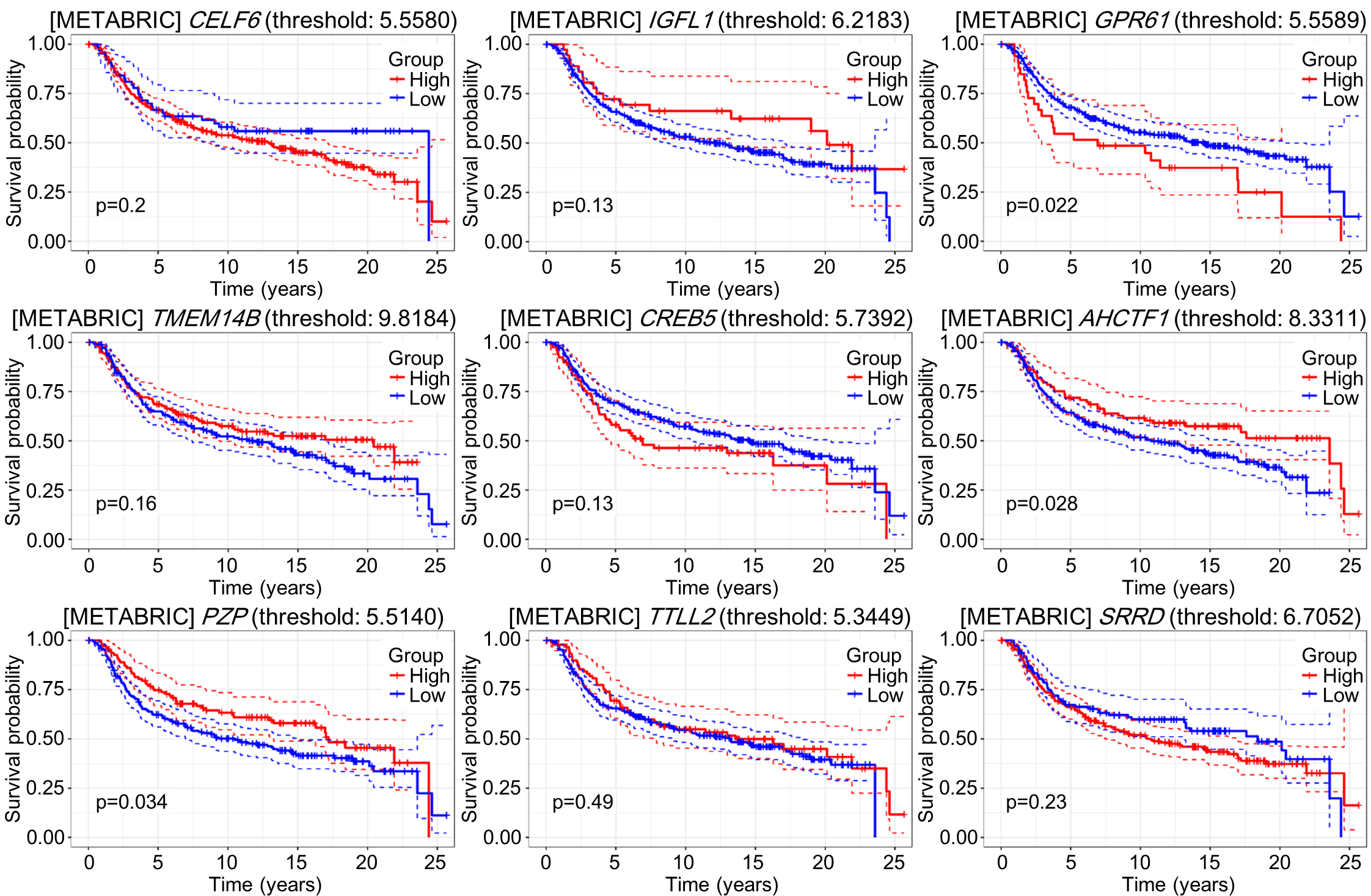

Supplement: S1 Fig — Analysis followed the procedure in Fig 3, but MaxStat cutoffs were re-estimated within METABRIC for each gene. Samples were dichotomized into high- (red) and low-expression (blue) groups using these cohort-specific cutoffs, and survival differences were assessed with the two-sided log-rank test. (TIF) [file pone.0348414.s001.tif]

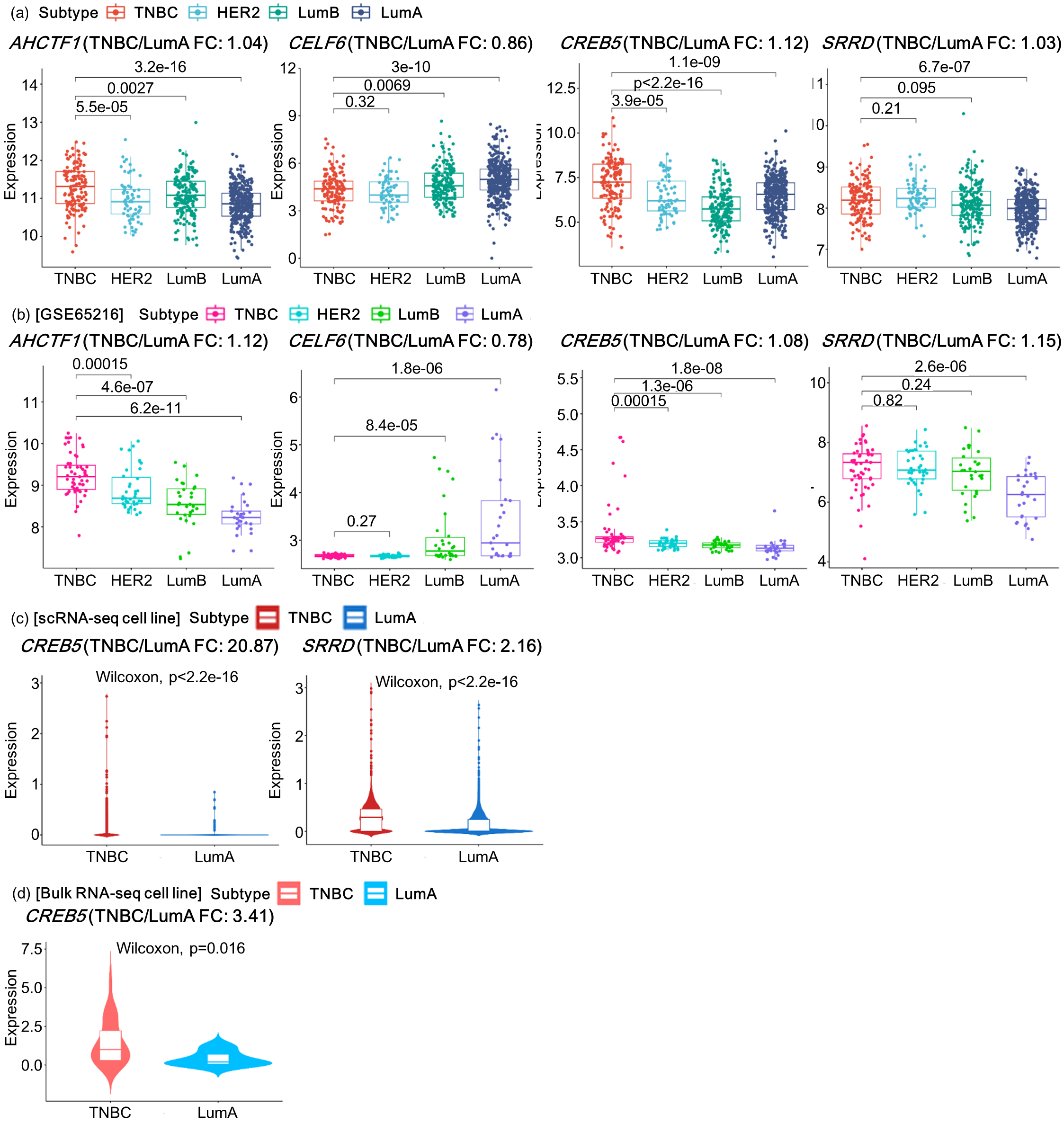

Supplement: S2 Fig — (a) TCGA-BRCA; (b) GSE65216; (c) cell line single cell RNA-seq; (d) cell line bulk RNA-seq. Fold change (FC) is the mean expression in TNBC divided by that in LumA. Differences between TNBC and LumA, LumB, and HER2 were tested using two-sided Wilcoxon rank-sum tests. (TIF) [file pone.0348414.s002.tif]
