# Supplementary material for: Development of predictive models for the prognosis of triple-negative breast cancer using multiple transcriptomic analyses
Source: PLoS One. 2026 May 4;21(5):e0348414. doi: 10.1371/journal.pone.0348414 (PMC13138617; doi:10.1371/journal.pone.0348414)
Supplement: S1 Table — NAC; Neoadjuvant chemotherapy. The p-values for continuous variable (age) and categorical variables were calculated using the Kruskal-Wallis test and the Chi-square test, respectively. (DOCX) [file pone.0348414.s003.docx]

**S1 Table. Clinicopathological characteristics of all patients in the TCGA-BRCA cohort.**

| Characteristics | TNBC  (N=143) | Luminal A  (N=386) | Luminal B  (N=186) | HER2  (N=69) | p-value |
| --- | --- | --- | --- | --- | --- |
| Age at diagnosis  [median (IQR)] | 54.0 (47.0 - 62.0) | 60.0 (49.0 - 68.0) | 58.0 (50.0 - 69.0) | 56.5 (50.7 - 64.0) | 0.0309 |
| History of NAC  [N (%)] |  |  |  |  | 0.3147 |
| No | 142 (99%) | 380 (98%) | 182 (98%) | 69 (100%) |  |
| Yes | 0 (0%) | 6 (2%) | 4 (2%) | 0 (0%) |  |
| AJCC stage [N (%)] |  |  |  |  | 2.0E-04 |
| I | 20 (14%) | 81 (22%) | 20 (11%) | 4 (6%) |  |
| II | 89 (62%) | 178 (47%) | 93 (50%) | 39 (57%) |  |
| III | 19 (13%) | 70 (19%) | 54 (29%) | 16 (23%) |  |
| IV | 3 (2%) | 5 (1%) | 4 (2%) | 2 (3%) |  |
| T staging [N (%)] |  |  |  |  | 2.3E-05 |
| T1 | 28 (20%) | 128 (34%) | 30 (16%) | 12 (17%) |  |
| T2 | 91 (64%) | 174 (46%) | 111 (60%) | 42 (61%) |  |
| T3 | 13 (9%) | 30 (8%) | 22 (12%) | 6 (9%) |  |
| T4 | 1 (1%) | 12 (3%) | 10 (5%) | 4 (6%) |  |
| N staging [N (%)] |  |  |  |  | 0.0139 |
| N0 | 84 (59%) | 167 (44%) | 71 (38%) | 25 (36%) |  |
| N1 | 35 (24%) | 121 (32%) | 62 (33%) | 23 (33%) |  |
| N2 | 10 (7%) | 37 (10%) | 29 (16%) | 10 (14%) |  |
| N3 | 5 (3%) | 20 (5%) | 11 (6%) | 6 (9%) |  |
| M staging [N (%)] |  |  |  |  | 0.7539 |
| M0 (Negative) | 132 (92%) | 338 (90%) | 167 (90%) | 62 (90%) |  |
| M1 (Metastasis) | 2 (1%) | 5 (1%) | 4 (2%) | 2 (3%) |  |
| Vital status |  |  |  |  | 0.1237 |
| Living | 125 (87%) | 339 (90%) | 155 (83%) | 54 (78%) |  |
| Deceased | 18 (13%) | 47 (13%) | 31 (17%) | 15 (22%) |  |

NAC; Neoadjuvant chemotherapy

The p-values for continuous variable (age) and categorical variables were calculated using the Kruskal-Wallis test and the Chi-square test, respectively.
