# Supplementary material for: Development of predictive models for the prognosis of triple-negative breast cancer using multiple transcriptomic analyses
Source: PLoS One. 2026 May 4;21(5):e0348414. doi: 10.1371/journal.pone.0348414 (PMC13138617; doi:10.1371/journal.pone.0348414)
Supplement: S2 Table — PFS: Progression Free Survival; DFS: Disease Free Survival. For the results, we utilized CoxPH as the machine learning algorithm and AUC as the prediction measure, both of which showed the highest predictive performance in Table 1. (DOCX) [file pone.0348414.s004.docx]

**S2 Table. The best model for each of PFS and DFS.**

| Response variable | No. of predictors | Model | Training (mean±s.d.) | Test (mean±s.d.) |
| --- | --- | --- | --- | --- |
| 5-year PFS | 9 | ADRA1B + ERRFI1 + AASS + CTNNA1 + RIMS1 + XG + COL4A2 + AQP5 + FMO5 | 0.9723±0.0119 | 0.9095±0.0722 |
| 10-year PFS | 9 | ADRA1B + ERRFI1 + AASS + PIGN + BVES + FMO5 + COL4A2 + AQP5 + CTNNA1 | 0.9751±0.0107 | 0.9248±0.0512 |
| 5-year DFS | 5 | TNK1 + LRRC58 + RIMS1 + PLD2 + XG | 0.8474±0.0427 | 0.7807±0.1385 |
| 10-year DFS | 5 | TNK1 + LRRC58 + PLD2 + XG + RIMS1 | 0.8533±0.0426 | 0.7889±0.1364 |

PFS: Progression Free Survival; DFS: Disease Free Survival

For the results, we utilized CoxPH as the machine learning algorithm and AUC as the prediction measure, both of which showed the highest predictive performance in Table 1.
